# Sine-Gordon Equation in (1+2) and (1+3) dimensions: Existence and Classification of Traveling-Wave Solutions

**DOI:** 10.1371/journal.pone.0124306

**Published:** 2015-05-28

**Authors:** Yair Zarmi

**Affiliations:** Jacob Blaustein institutes for Desert Research, Ben-Gurion University of the Negev, Midreshet Ben-Gurion, 8499000, Israel; University of Calgary, CANADA

## Abstract

The (1+1)-dimensional Sine-Gordon equation passes integrability tests commonly applied to nonlinear evolution equations. Its kink solutions (one-dimensional fronts) are obtained by a Hirota algorithm. In higher space-dimensions, the equation does not pass these tests. Although it has been derived over the years for quite a few physical systems that have nothing to do with Special Relativity, the Sine-Gordon equation emerges as a non-linear relativistic wave equation. This opens the way for exploiting the tools of the Theory of Special Relativity. Using no more than the relativistic kinematics of tachyonic momentum vectors, from which the solutions are constructed through the Hirota algorithm, the existence and classification of *N*-moving-front solutions of the (1+2)- and (1+3)-dimensional equations for all *N* ≥ 1 are presented. In (1+2) dimensions, each multi-front solution propagates rigidly at one velocity. The solutions are divided into two subsets: Solutions whose velocities are lower than a limiting speed, *c* = 1, or are greater than or equal to *c*. To connect with concepts of the Theory of Special Relativity, *c* will be called “the speed of light.” In (1+3)-dimensions, multi-front solutions are characterized by spatial structure and by velocity composition. The spatial structure is either planar (rotated (1+2)-dimensional solutions), or genuinely three-dimensional – branes. Planar solutions, propagate rigidly at one velocity, which is lower than, equal to, or higher than *c*. Branes must contain clusters of fronts whose speed exceeds *c* = 1. Some branes are “hybrids”: different clusters of fronts propagate at different velocities. Some velocities may be lower than *c* but some must be equal to, or exceed, *c*. Finally, the speed of light cannot be approached from within the subset of slower-than-light solutions in both (1+2) and (1+3) dimensions.

## Introduction

### 1.1 Open problems concerning the Sine-Gordon equation

SG*n*, the Sine-Gordon equation in (1+*n*)-dimensions,

$$\partial_{\mu }\partial^{\mu }u+\text{sin}\;u\equiv \left\{\partial_{t}{}^{2}-\left(\partial_{x_{1}}{}^{2}+\ldots +\partial_{x_{n}}{}^{2}\right)\right\}u+\text{sin}\;u=0,\;\mu =0,1,..,n,\;n=1,2,3\;,$$ (1)

has attracted wide attention over the years in the description of classical and quantum mechanical phenomena [1–9], and within the framework of quantum-field theory [9–17]. In the study of specific physical systems, the spatial derivatives in Eq (1) are multiplied by *c* ^2^, where *c* is the velocity of wave propagation in the linearized equation. This velocity is determined by the physical parameters that characterize each system. Eq (1) is written in terms of scaled coordinates, so that *c*, the limiting speed of propagation of signals is equal to unity.

Hirota provided the algorithm for the construction of the kink solutions (moving fronts) of the (1+1) dimensional equation [18], which was then shown to be integrable [19]. In contradistinction, it has been known for decades that the (1+2) dimensional equation does not pass integrability tests that are traditionally applied to nonlinear evolution equations. It is not integrable within the framework of the Inverse-Scattering formalism [20] and it does not have the properties required for integrability in a Painlevé analysis [21–24]. Furthermore, in (1+2) dimensions, whereas single- and two-front solutions could be constructed through the Hirota algorithm, the attempt to construct a three-front solution encountered an obstacle [25]: For a three-front solution to exist, the parameter sets (three parameters for each front) from which the solution is constructed had to obey a constraint. A different constraint was found in the construction of multi-front solutions of the (1+3) dimensional Sine-Gordon equation [26].

Over the years, quite a few works (see, e.g., Refs. [27–32]) have approached the issue of the construction of travelling-wave solutions in (1+2) or (1+3) dimensions. Still, a complete algorithm is presented only in Refs. [25, 26]. These solutions, often called “soliton solutions”, actually, represent moving fronts. In (1+1) dimensions they have been also called “kinks”. For visualization purposes, one often uses the current density, *J*_*μ*_ = *∂*_*μ*_ *u*, as the latter does display solitons.

Although the Sine-Gordon equation was derived for quite a few physical systems that have nothing to do with Special Relativity, the equation itself emerges as a non-linear relativistic wave equation. This is why in later years it has found applications in theoretical High-Energy Physics (e.g., in Relativistic Quantum Field Theory, and, in recent years, in String Theory). This characteristic of the equation calls for the exploitation of the tools of the Theory of Special Relativity (see, e.g., ref. [33]) in the analysis of its solutions.

The seed for this approach was sawn [5, 8] *before* the Hirota algorithm for the construction of multi-front solutions [18] was known. The physical system for which the equation was applied was that of a nonlinear electrical circuit as a model for the Josephson Junction. The Lorentz invariance of the equation, and its single-front solution were discussed in ref. [8]. However, since then, this idea was not pursued in the literature. Specifically, the literature involved in the construction of traveling-wave solutions of SG*n* for *n* = 2 and 3 has not exploited useful properties of the parameters that multi-front solutions are constructed from in the Hirota algorithm. Each front is associated with a set of (1+*n*) parameters, which may be viewed as the components of a tachyonic momentum vector (a space-like vector with (mass)^2^ = -1) in the (1+*n*)-dimensional space. In this paper, the properties under Lorentz transformations of these vectors are exploited, allowing for a demonstration of a physical nature of the existence, richness and classification of *N*-front solutions, for any *N* ≥ 1, of SG*n* for *n* > 1. To ensure that it is clear that the metric for a scalar product of vectors in the space is (+1,-1,… -1) rather than the (+1,+1,…,+1) metric employed in Euclidean space, the Special-Relativity term “Minkowski space” will be used for the (1+*n*)-dimensional space.

Section 1.2 presents a review of the construction of front solutions of SG1 (kinks) through the Hirota algorithm [18]. Section 1.3 summarizes the results presented in the paper. Section 2 is devoted to a review of the properties under Lorentz transformations of tachyonic momentum vectors. These properties are the basis for the arguments used in the rest of the paper. Sections 3 and 4 discuss the construction of moving-front solutions in, respectively, (1+2) and (1+3) dimensions. Section 5 presents an invariance property of the multi-front solutions of SG3, which propagate at velocities that are lower than *c*. Section 6 presents some numerical examples of solutions. Section 7 briefly summarizes the results for Eq (1), with the sign of the sin *u*-term changed to a (-) sign.

#### 1.1.1 Motivation

The interest in front solutions of the Sine-Gordon equation in more than one space dimension goes beyond the mere challenge of finding them. The (1+2) dimensional equation is relevant, for example, in the study of extended Josephson junctions, and has potential application in the study of stretching and folding of elastic sheets and in the structure of DNA chains.

### 1.2 Moving-front (kink) solutions of Sine-Gordon equation in (1+1) dimensions—A review

#### 1.2.1 Construction of solutions

The first step in the Hirota algorithm [18] for the construction of the moving solutions of SG1 is a transformation, originally proposed in the cases of one- and two-front solutions [7,8]:

$$u\left(x;P\right)=4\;{\text{tan}}^{-1}\left[g\left(x;P\right)/f\left(x;P\right)\right]\;.$$ (2)

In Eq (2),

$$P\equiv \left\{p^{\left(1\right)},p^{\left(2\right)},\ldots ,p^{\left(N\right)}\right\}\;.$$ (3)

*x* and *p* ^(*i*)^ are coordinate and momentum vectors in (1 + 1) dimensions.

The functions *g*(*x*;*P*) and *f*(*x*;*P*) are given by:

$$g\left(x;P\right)=\sum_{\begin{array}{c} 1\leq n\leq N\\ n\;odd \end{array}}\left(\sum_{1\leq i_{1}<\cdots <i_{n}\leq N}\left\{\prod_{j=1}^{n}\varphi \left(x;p^{\left(i_{j}\right)}\right)\prod_{i_{l}<i_{m}}V\left(p^{\left(i_{l}\right)},p^{\left(i_{m}\right)}\right)\right\}\right)\;,$$ (4)

$$f\left(x;P\right)=1+\sum_{\begin{array}{c} 2\leq n\leq N\\ n\;even \end{array}}\left(\sum_{1\leq i_{1}<\cdots <i_{n}\leq N}\left\{\prod_{j=1}^{n}\varphi \left(x;p^{\left(i_{j}\right)}\right)\prod_{i_{l}<i_{m}}V\left(p^{\left(i_{l}\right)},p^{\left(i_{m}\right)}\right)\right\}\right)\;,$$ (5)

$$\varphi \left(x;p^{\left(i\right)}\right)=e^{p^{\left(i\right)}{}_{\mu }x^{\mu }+\delta_{i}}\;,$$ (6)

$$p_{\mu }^{\left(i\right)}p^{\left(i\right)\;\mu }=-1\;,$$ (7)

and

$$V\left(p,p'\right)=\frac{{\left(p-p'\right)}_{\mu }{\left(p-p'\right)}^{\mu }}{{\left(p+p'\right)}_{\mu }{\left(p+p'\right)}^{\mu }}=\frac{1+p_{\mu }p{'}^{\mu }}{1-p_{\mu }p{'}^{\mu }}\;.$$ (8)

Finally, *N* is the number of fronts (kinks) displayed by the solution. The *N* solitons show up in the current density:

$$J_{\mu }=\partial_{\mu }u\;.$$ (9)

In Eq (6), *δ*_*i*_ is a constant arbitrary phase. Eq (7) states that the set of parameters associated with each front is a space-like vector with invariant mass^2^ = -1. i.e., a tachyonic vector.

#### 1.2.2 Properties of (1+1)-dimensional solutions

Most of the properties of front solutions of SG1 (kinks) are shared by the solutions of SG2 and SG3. (In the latter equations, *x* and *p* ^(*I*)^ are vectors in, respectively, (1 + 2) and (1 + 3) dimensions.) When a difference between SG1 and SG2 or SG3 exists, it is pointed out explicitly.

1. The solution, *u*(*x*;*P*), is a function of Lorentz scalars only. As a result, it is invariant under Lorentz transformations that are applied simultaneously to the coordinate vector, *x*, and the momentum vectors. This was first pointed out in the case of the single-front solution in Ref. [5].
2. Depending on the free phases in Eq (6), a solution with *N* ≥ 3 fronts will exhibit in the (1+*n*)-dimensional Minkowski space anywhere from one junction, when all phases are sufficiently small, up to *N*(*N*-1)/2 junctions, when the phases are sufficiently large. It is more convenient to see this in the solitons displayed by the current density (see Eq (9)). Sufficiently far from all junctions, *J*_*μ*_ splits up into a sum of single solitons, each associated with one of the momenta, *p* ^(*i*)^, used in Eqs (2)–(8). (For an example, see Section 6.)
3. In an *N*-front solution, all pairs of momentum vectors obey *p* ^(*i*)^ ≠ ±*p* ^(*j*)^ for *i* ≠ *j*. If equality holds, then the *N*-front solution degenerates into a solution with a smaller number of fronts.
4. Owing to Eq (7), the velocity of each individual front (be it a single-front solution, or one front in a multi-front solution, away from front junctions) is lower than the speed of light (*c* = 1). This is a consequence of the fact that a single-front solution depends on one exponential:
  $$u(x;p)=4\;{\text{tan}}^{-1}[e^{p_{0}t-p_{1}x+\delta }]\;.$$ (10)
  Consequently, the velocity, *v*, of propagation of a single front is bounded by:
  $$\left|v\right|=\left|p_{0}/p_{1}\right|=\sqrt{p_{1}{}^{2}-1}/\left|p_{1}\right|\leq c=1\;.$$ (11)
  Clearly, the fact that *c* = 1 is a consequence of the use of scaled coordinates. For example, in the derivation of the Sine-Gordon equation as the continuum limit of the description of the motion of a dislocation in a periodic one-dimensional lattice [1], *c*, the velocity of waves in the linearized equation, is determined by the strength of the periodic potential in the lattice and the period of the potential. In the description of the structure of DNA molecules, it may be viewed as the limiting velocity of acoustic waves. As in different applications the limiting velocity, *c*, has a different interpretation, and in order to connect with tools of Special Relativity, *c* = 1, the limiting velocity that characterizes the solutions in the scaled coordinates used in Eq (1), will be called “the speed of light”.
  The fact that *v*, the speed of propagation of an individual front, is lower than *c* means that a Lorentz boost with a velocity equal to *v* transforms the front to a rest frame. The solution and the associated momentum vector are transformed as follows [5]:
  $$\left\{p_{0},p_{1}\right\}\to \left\{0,\pm 1\right\}\;\Rightarrow \;u\left(x;p\right)\to 4\;{\text{tan}}^{-1}\left[e^{\pm x_{1}+\delta }\right]\;.$$ (12)
  Thus, in its rest fame, an individual front is static (stationary and time-independent). However, a transformation of any multi-front solution of SG1 to a rest frame does not exist, because each front propagates along the *x*-axis at a different velocity.
  The situation is different in the case of the slower-than-light multi-front solutions of SG2 and SG3. Each of these solutions has a planar structure and propagates rigidly in the plane it defines at a constant velocity. Hence, it can be Lorentz transformed to a rest frame, in which it is static.
5. A Hirota-type single-kink solution of SG1 propagates at a velocity that is lower than *c*, and is stable. A single-wave solution, not a Hirota front, which propagates at velocities that exceed *c* also exists and is unstable. These statements were demonstrated through a Sturm-Liouville analysis [8]. The stability of Hirota-type solutions in higher space dimensions is still an open problem, as there is no extension of the Sturm-Liouville theory beyond one dimension.

### 1.3 Extension of Eq (1) to higher space dimensions

Sections 3 and 4 present the construction of *N*-front solutions of Eq (1) through the Hirota algorithm in (1+*n*) dimensions for respectively, *n* = 2 and 3, for all *N* ≥ 1. It will be shown that:

1. In solutions of SG2 with *N* ≥ 3 fronts, (*N* − 2) of the momentum vectors in Eqs (2)–(8) are linear combinations of just two of them [34]. Each multi-front solution of SG2 propagates rigidly at a constant velocity, *v*, in the plane defined by the two “basis” vectors. The solutions are divided into two unconnected subsets, with *v* < *c* = 1, and *v* ≥ c. Slower-than-light solutions can be Lorentz-transformed to a rest frame, in which they are static (stationary and time-independent).
2. The multi-front solutions of SG3 are divided into four subsets. Two subsets have a planar structure. They are the space-rotated (1+2)-dimensional solutions. The solutions in the third and fourth subsets are genuinely three-dimensional structures—branes. Solutions in the third subset propagate rigidly at the speed of light. The fourth subset contains “hybrid” solutions, in which some clusters of fronts may propagate rigidly at velocities that are lower than *c*, but there are clusters that propagate at velocities that are greater than, or equal to *c*. Different clusters may have different velocities. One front may participate in more than one cluster.
3. It is impossible to reach the speed of light as a limit of slower-than-light solutions.

### 1.4 Methods

The construction of solutions of Eq (1) in (1+2) and (1+3) dimensions follows the steps presented in Eqs (2)–(8). The only change is that now, the position vector, *x*, and the momentum vectors, *p* ^(*i*)^, used in the construction are vectors in, correspondingly, the (1+2) and (1+3)-dimensional Minkowski space.

The main tool exploited in the demonstration of the existence of solutions, their construction and classification is the properties of the momentum vectors under Lorentz transformations in Minkowski space in (1+2), or (1+3) dimensions. These properties are discussed in Section 2, and exploited in Sections 3–7. The fact that the momentum vectors are tachyonic (having (invariant mass)^2^ = -1) plays a crucial role in the characteristics of the solutions. For example, this property ensures that each individual front, be it a single-front solution, or one front in a multi-front solution, but far away from the region in space, in which fronts collide, propagates at a constant velocity *v*, that is lower than the speed of light (*c* = 1). Still, clusters of fronts may propagate rigidly at velocities that are lower than *c*, or exceed *c*. The existence of three-front solutions is discussed explicitly. The steps in the demonstration of the existence of four-front solutions are reviewed, and the existence of solutions with *N* > 4 fronts follows by induction.

Requiring that *u*(*t*,*x*), constructed via Eqs (2)–(8), be a solution of Eq (1) in (1+2) or (1+3) dimensions yields constraints that the momentum vectors have to obey. These constraints are discusses in Sections 2, 3 and 4. They affect the physical characteristics of the multi-front solutions, namely, their spatial structure and velocity profiles. For example, the constraint on the momenta in (1+2) dimensions forces a multi-front solution to propagate rigidly in the *x*-*y* plane at a constant velocity, which may be either lower than, or exceed *c*.

## Relativistic Analysis of Space-Like Momentum-Vectors

Owing to the space-like nature of the momentum vectors, from which Hirota-type moving-front solutions are constructed (see Eq (7)), each individual front propagates at a velocity that is lower than *c*. However, in more than one space dimension, the velocity of a cluster of fronts may be lower than, equal to, or exceed *c* = 1. This counter-intuitive phenomenon is a direct consequence of the tachyonic nature of the momentum vectors. It does not exist in the case of time-like vectors, reviewed in Section 7.

To classify *N*-front solutions, one needs to find the conditions under which clusters of two or more fronts propagate rigidly at one velocity, and what that velocity is. In particular, one needs to know if it is possible to transform a cluster of fronts, or a whole multi-front solution, to a rest frame, in which it is static: stationary and time independent. Such a cluster must be moving rigidly as a whole at a velocity that is lower than *c*, so that it can be Lorentz-transformed to its rest frame. Concurrently, to ensure that the static solution is time-independent, the momentum vectors associated with all fronts in the cluster ought to be transformed to a purely space-like form in the Lorentz frame, in which the solution is static:

$$p^{\left(i\right)}=\{p_{0}^{\left(i\right)},{\vec{p}}^{\left(i\right)}\}\to \{0,{\vec{q}}^{\left(i\right)}\},\;({\vec{q}}^{\left(i\right)}\cdot {\vec{q}}^{\left(i\right)}=1)\;.$$ (13)

In Eq (13), ${\vec{q}}^{\left(i\right)}$ is a unit vector in the *n*-dimensional Euclidean space.

A Lorentz transformation in (1+3) dimensions with a boost velocity $\vec{v}$ is represented by:

$$L=\left(\begin{array}{cccc} \gamma & -\gamma \;\beta_{x} & -\gamma \;\beta_{y} & -\gamma \;\beta_{z}\\ -\gamma \;\beta_{x} & 1+\left(\gamma -1\right)\frac{\beta_{x}{}^{2}}{\beta^{2}} & \left(\gamma -1\right)\frac{\beta_{x}\beta_{y}}{\beta^{2}} & \left(\gamma -1\right)\frac{\beta_{x}\beta_{z}}{\beta^{2}}\\ -\gamma \;\beta_{y} & \left(\gamma -1\right)\frac{\beta_{x}\beta_{y}}{\beta^{2}} & 1+\left(\gamma -1\right)\frac{\beta_{y}{}^{2}}{\beta^{2}} & \left(\gamma -1\right)\frac{\beta_{y}\beta_{z}}{\beta^{2}}\\ -\gamma \;\beta_{z} & \left(\gamma -1\right)\frac{\beta_{x}\beta_{z}}{\beta^{2}} & \left(\gamma -1\right)\frac{\beta_{y}\beta_{z}}{\beta^{2}} & 1+\left(\gamma -1\right)\frac{\beta_{z}{}^{2}}{\beta^{2}} \end{array}\right)\;.$$ (14)

In Eq (14),

$$\vec{\beta }=\left\{\beta_{x},\beta_{y},\beta_{z}\right\}=\left\{v_{x},v_{y},v_{z}\right\}/c\;,\;\gamma =1/\sqrt{1-{\vec{\beta }}^{2}}\;.$$ (15)

In (1+2) dimensions, the matrix is reduced to its top leftmost 3×3 minor, and in (1+1) dimensions—to the 2×2 minor.

A single vector that obeys Eq (7) can be always Lorentz-transformed to the form given in Eq (13). In (1+3) dimensions, there will be a two-parameter family of transformations that will achieve this, as only one of the components of $\vec{\beta }$ can be determined. In (1+2) dimensions, this will be a one parameter family, and in (1+1) dimensions-a unique transformation.

Next, consider a pair of two vectors, *p* ^(1)^ ≠ ±*p* ^(2)^. In (1+1) dimensions, no transformation can transform both vectors to the form given in Eq (13). There is only one free parameter, *β*_*x*_, and there are two quantities, $p_{0}^{\left(i\right)}$, *i* = 1,2 that have to be transformed to zero.

There is no need to discuss the (1+3)-dimensional case, because the space components of the two vectors define a plane. Hence, one can first rotate the two vectors so that their *z*-components vanish, and reduce the system to (1+2)-dimensions:

$$p^{\left(i\right)}=\left\{p_{0}^{\left(i\right)},p_{x}^{\left(i\right)},p_{y}^{\left(i\right)}\right\}\;\left(i=1,2\right)\;.$$ (16)

The boost parameters, *β*_*x*_ and *β*_*y*_ of Eqs (14) and (15), required for the transformed vectors to have vanishing time components, as in Eq (13) are:

$$\beta_{x}=-\frac{p_{0}^{\left(1\right)}\;p_{y}^{\left(2\right)}-p_{0}^{\left(2\right)}\;p_{y}^{\left(1\right)}}{p_{x}^{\left(1\right)}\;p_{y}^{\left(2\right)}-p_{x}^{\left(2\right)}\;p_{y}^{\left(1\right)}}\;,\;\beta_{y}=\frac{p_{0}^{\left(1\right)}\;p_{x}^{\left(2\right)}-p_{0}^{\left(2\right)}\;p_{x}^{\left(1\right)}}{p_{x}^{\left(1\right)}\;p_{y}^{\left(2\right)}-p_{x}^{\left(2\right)}\;p_{y}^{\left(1\right)}}\;.$$ (17)

For the transformation to be feasible, its velocity must be lower than *c*. Hence, the magnitude of the vector $\vec{\beta }$ must be smaller than 1. Using Eqs (17) and (7), this requirement yields:

$$1-\beta_{x}{}^{2}-\beta_{y}{}^{2}=\frac{1-{\left(p^{\left(1\right)}\cdot p^{\left(2\right)}\right)}^{2}}{{\left(p_{x}^{\left(1\right)}\;p_{y}^{\left(2\right)}-p_{x}^{\left(2\right)}\;p_{y}^{\left(1\right)}\right)}^{2}}>0\;.$$ (18)

Thus, for a pair of vectors that obey Eq (7) to be Lorentz-transformable to the form given in Eq (13), its scalar product in Minkowski space must obey

$$\left|p^{\left(1\right)}\cdot p^{\left(2\right)}\right|<1\;.$$ (19)

If the inequality is inverted,

$$\left|p^{\left(1\right)}\cdot p^{\left(2\right)}\right|\geq 1\;,$$ (20)

then there is no velocity lower than *c* that can yield the desired Lorentz transformation.

In the case of vectors that obey Eq (19) (corresponding to a pair of fronts that move rigidly at a velocity that is lower than *c* = 1), the limit of equality,

$$p^{\left(1\right)}\cdot p^{\left(2\right)}\to \pm 1\;,$$ (21)

is reached with

$$p^{\left(2\right)}\to \mp p^{\left(1\right)}\;.$$ (22)

This can be shown by first transforming *p* ^(1)^ and *p* ^(2)^ to the form given in Eq (13). Eq (21) is then expressed in terms of the two-dimensional unit vectors as:

$$p^{\left(1\right)}\cdot p^{\left(2\right)}=-{\vec{q}}^{\left(1\right)}\cdot {\vec{q}}^{\left(2\right)}=-\text{cos}\left({\vec{q}}^{\left(1\right)},{\vec{q}}^{\left(2\right)}\right)\to \pm 1\;.$$ (23)

$\text{cos}\left({\vec{q}}^{\left(1\right)},{\vec{q}}^{\left(2\right)}\right)$ is the cosine of the angle between the two unit vectors. The limit is obtained by:

$${\vec{q}}^{\left(2\right)}\to \mp {\vec{q}}^{\left(1\right)}\;,$$ (24)

from which Eq (22) follows in any moving frame through a Lorentz transformation.

In contrast, when Eq (20) holds, the previous argument does not apply; one cannot transform the two vectors to the form of Eq (13) simultaneously. The limit of Eq (21) then consists of a continuum of vectors in (1+2) or (1+3) dimensions. A simple way to see this, is to Lorentz transform one of the vectors, say *p* ^(1)^, as in Eq (13) (this *is* possible), with *p* ^(2)^ transformed as follows:

$$p^{\left(1\right)}\to \{0,{\vec{q}}^{\left(1\right)}\}\;({\vec{q}}^{(1)}\cdot {\vec{q}}^{\left(1\right)}=1)\;,\;p^{\left(2\right)}\to \{q_{0}^{\left(2\right)},{\vec{q}}^{\left(2\right)}\}\;.$$ (25)

(Note that, in Eq (25), ${\vec{q}}^{\left(2\right)}$ is *not* a unit vector!) Eq (21) then becomes:

$$p^{\left(1\right)}\cdot p^{\left(2\right)}=\left|{\vec{q}}^{\left(2\right)}\right|\;\text{cos}\left({\vec{q}}^{\left(1\right)},{\vec{q}}^{\left(2\right)}\right)=\pm 1\;.$$ (26)

Eqs (26) and (7) yield:

$$\left|{\vec{q}}^{\left(2\right)}\right|=\frac{1}{\left|\text{cos}\left({\vec{q}}^{\left(1\right)},{\vec{q}}^{\left(2\right)}\right)\right|}\;,\;q_{0}^{\left(2\right)}=\pm \text{tan}\left({\vec{q}}^{\left(1\right)},{\vec{q}}^{\left(2\right)}\right)\;.$$ (27)

Thus, there is a continuum of pairs of vectors for which the limit of Eq (21) can be achieved.

In (1+3) dimensions, there is another possibility, of simultaneously Lorentz-*transforming three space-like vectors*, which obey Eq (7), to the form given by Eq (13). Applying the Lorentz transformation of Eq (14) to the three vectors:

$$p^{\left(i\right)}=\left\{p_{0}^{\left(i\right)},p_{x}^{\left(i\right)},p_{y}^{\left(i\right)},p_{z}^{\left(i\right)}\right\}\;,\;\left(i=1,2,3\right)\;,$$ (28)

and requiring that the transformed vectors be of the form given in Eq (13), one finds that the parameters of the transformation have to be:

$$\beta_{x}=\frac{\Delta_{x}}{\Delta_{0}}\;,\;\beta_{y}=\frac{\Delta_{y}}{\Delta_{0}}\;,\;\beta_{z}=\frac{\Delta_{z}}{\Delta_{0}}\;.$$ (29)

In Eq (29),

$$\begin{array}{c} \Delta_{x}=\left|\begin{array}{ccc} p_{0}^{\left(1\right)} & p_{y}^{\left(1\right)} & p_{z}^{\left(1\right)}\\ p_{0}^{\left(2\right)} & p_{y}^{\left(2\right)} & p_{z}^{\left(2\right)}\\ p_{0}^{\left(3\right)} & p_{y}^{\left(3\right)} & p_{z}^{\left(3\right)} \end{array}\right|\;,\;\Delta_{y}=\left|\begin{array}{ccc} p_{0}^{\left(1\right)} & p_{z}^{\left(1\right)} & p_{x}^{\left(1\right)}\\ p_{0}^{\left(2\right)} & p_{z}^{\left(2\right)} & p_{x}^{\left(2\right)}\\ p_{0}^{\left(3\right)} & p_{z}^{\left(3\right)} & p_{x}^{\left(3\right)} \end{array}\right|\;,\;\Delta_{z}=\left|\begin{array}{ccc} p_{0}^{\left(1\right)} & p_{x}^{\left(1\right)} & p_{y}^{\left(1\right)}\\ p_{0}^{\left(2\right)} & p_{x}^{\left(2\right)} & p_{y}^{\left(2\right)}\\ p_{0}^{\left(3\right)} & p_{x}^{\left(3\right)} & p_{y}^{\left(3\right)} \end{array}\right|\\ \Delta_{0}=\left|\begin{array}{ccc} p_{x}^{\left(1\right)} & p_{y}^{\left(1\right)} & p_{z}^{\left(1\right)}\\ p_{x}^{\left(2\right)} & p_{y}^{\left(2\right)} & p_{z}^{\left(2\right)}\\ p_{x}^{\left(3\right)} & p_{y}^{\left(3\right)} & p_{z}^{\left(3\right)} \end{array}\right| \end{array}\;.$$ (30)

When *Δ*_0_ ≠ 0, for the transformation to be feasible, the magnitude of $\vec{\beta }$ must be smaller than 1. If *Δ*_0_ = 0, there is no solution for $\vec{\beta }$ unless *Δ*_*x*_, *Δ*_*y*_, and *Δ*_*z*_ vanish as well. As a result, the three vectors must be linearly dependent. Hence, the system can be rotated to (1+2) dimensions. This point will be of relevance in the classification of multi-front solutions in (1+3) dimensions.

## Moving-Front Solutions in (1+2) Dimensions

### 3.1 Brief summary

As will be shown in the following, in the case of *N* ≥ 3 fronts, only two of the momentum vectors are independent. The remaining vectors are given as linear combinations of the two “basis” vectors, *p* ^(1)^ and *p* ^(2)^. If this pair of vectors obeys Eq (19), then the coefficients *V*(*p* ^(*i*)^,*p* ^(*j*)^) in Eqs (4), (5) and (8) are all positive. Hence, *f*(*x*;*P*) of Eq (5) does not vanish anywhere. The resulting multi-front solution, *u*(*x*;*P*), varies (possibly, several times) over the range [0, 2 *π*]. Concurrently, the solution propagates at a velocity that is lower than *c* = 1.

If *p* ^(1)^ and *p* ^(2)^ obey Eq (20), then *f*(*x*;*P*) of Eq (5) vanishes on some manifold (a line in (1+1) dimensions, a plane in (1+2) dimensions). The resulting multi-front solution, *u*(*x*;*P*), varies over the range [0, 2 *N π*], where *N* is the number of fronts. Concurrently, the solution propagates at a velocity that is greater than, or equal to *c*.

### 3.2 Single- and two-front solutions of SG2

The solution procedure of SG2 is rather cumbersome, and best implemented with the aid of symbolic manipulation software. (MATHEMATICA has been used in this study). One begins by substituting Eqs (2)–(5) in

$$Q=\left(\partial_{\mu }\partial^{\mu }u+\text{sin}\;u\right){\left(f{\left(x;P\right)}^{2}+g{\left(x;P\right)}^{2}\right)}^{2}\;.$$ (31)

Eqs (6)–(8) ensure that *Q* vanishes for the single-and two-front solutions.

The single-front solution propagates at a velocity that is lower than *c* = 1. The situation in the case of the two-front solution is different. Constructing this solution through Eqs (2)–(8) with two momentum vectors, *p* ^(1)^ and *p* ^(2)^, finds that two-front solution obeys the following identity:

$$u\left(t,\vec{x}+\vec{\beta }t\right)=u\left(t=0,\vec{x}\right)\;.$$ (32)

In Eq (32), the time- and space-parts of *x*, the position vector in (1+2) dimensions, have been written out explicitly. The components of the velocity vector, $\vec{\beta }$, are given in Eq (17). Thus, the two-front solution propagates at a velocity that is either lower than *c* or exceeds *c*, depending whether $\left|\vec{\beta }\right|<1$ or $\left|\vec{\beta }\right|\geq 1$, respectively. This requires that Eq (19) or Eq (20), respectively, holds for the two momentum vectors, from which the solution is constructed.

### 3.3 Three-front solution of SG2

Repeating the procedure delineated above in the case of three-front solutions of SG2, after implementing Eqs (6)–(8), the quantity *Q* of Eq (31) remains proportional to (Δ_*z*_)^2^, with Δ_*z*_ defined in Eq (30). For a three-front solution to exist, Δ_*z*_ must, therefore, vanish. This requirement was first observed by Hirota [25]. It means that a three-front solution exists only if one of the three momentum vectors, say *p* ^(3)^, is a linear combination of the other two vectors 34]. This also means that the solution has a planar structure, as the three momentum vectors lie in a plane.

### 3.4 *N* > 3 front solutions of SG2

The proof that, in solutions with *N* > 3 momentum vectors, (*N−*2) of the vectors must be linear combinations of just two of them is cumbersome but straightforward. To show how the proof goes, consider the case of *N* = 4. One repeats the construction procedure delineated above through Eqs (2)–(8). Among the remaining monomials in *Q* of Eq (31), one considers the collection of monomials that do not $\varphi \left(x;p^{\left(i\right)}\right)=e^{p^{\left(i\right)}\cdot x}$ for some value of 1 ≤ *i* ≤ 4. This collection is just *Q* in the three-front case (i.e., as if the front associated with *p* ^(*i*)^ did not exist). For this three-front part to vanish, the remaining three vectors, *p* ^(*j*)^, 1 ≤ *j* ≠ *i* ≤ 4, must be linearly dependent, as found by Hirota [25] and reviewed in Section 3.3. This must hold for any1 ≤ *i* ≤ 4. Hence, of the four vectors, two must be linear combinations of the other two. The proof for any *N* > 3 is by induction. The Hirota condition [25] is found to apply to each triplet of vectors that can be formed from the *N* momentum vectors. Hence, (*N–*2) of the vectors must be linear combinations of just two vectors 34]:

$$p^{\left(i\right)}=\mu_{i}\;p^{\left(1\right)}+\nu_{i}\;p^{\left(2\right)}\;\left(3\leq i\leq N\right)\;.$$ (33)

### 3.5 Properties of (1+2)-dimensional solutions

As a result of Eq (33), the velocity of a solution with *N* ≥ 2 fronts is determined by the velocity required for the transformation of the two “basis” vectors (the choice of which is arbitrary), *p* ^(1)^ and *p* ^(2)^, from which all other momentum vectors are constructed, to the form given in Eq (13).

If these two vectors obey Eq (19), then a Lorentz transformation with a velocity, given in Eq (17), and lower than *c*, transforms the two vectors to the form given in Eq (13). Hence, the solution propagates rigidly at that velocity, and is transformed by the transformation to a rest frame, in which it is static: stationary and time-independent. If the two basis vectors obey Eq (20), then the velocity of Eq (17) exceeds *c*, corresponding to a solution that propagates rigidly at a velocity, *v* ≥ *c*. Thus, the (1+2)-dimensional solutions are divided into two subsets: solutions that propagate as a whole at *v* < *c* and at *v* ≥ *c*.

The inclusion of the case of solutions whose velocity is *v* = *c* in the second subset is not arbitrary. If the two basis vectors, *p* ^(1)^ and *p* ^(2)^, obey Eq (19), then the limit of equality, Eq (21), is achievable only through Eq (22). However, direct substitution in Eqs (2)–(8) yields that, in this limit, a slower-than-light solution degenerates into a slower-than-light solution with a smaller number of fronts. If the (+) sign holds in Eq (22) then the coefficient *V*(*p* ^(1)^,*p* ^(2)^) of Eq (8) vanishes, causing the solution to degenerate into a slower-than-light solution with (*N*-1) fronts. If the (-) sign holds in Eq (22), then *V*(*p* ^(1)^,*p* ^(2)^) becomes infinite, causing the solution to degenerate into a slower-than-light solution with (*N*-2) fronts. *In summary*, *the speed of light cannot be achieved from within the subset of slower-than-light solutions*.

The situation is different in the case of solutions constructed with basis vectors that obey Eq (20). The front pairs then propagate rigidly at velocities *v* ≥ *c*. The discussion that leads to Eqs (25)–(27) shows that the limit *v* = *c* is included in this subset of solutions.

## Front Solutions in (1+3) Dimensions

### 4.1 Single- and two-front solutions of SG3

The single- and two-front solutions of SG3 are readily generated by the Hirota algorithm [18]. As in the case of the solutions of SG2, the single-front solution propagates at a velocity that is lower than *c*, whereas the velocity of the two-front solution is lower than, equal to, or higher than *c*, depending on whether the two associated momentum vectors obey, respectively, Eq (19) or Eq (20). It is nothing but a spatially rotated (1+2)-dimensional solution.

### 4.2 *N* ≥ 3 front solutions of SG3

Let us begin with the three-front solution. After having implemented Eqs (2)–(8), one finds that for the solution to exist (namely, for *Q* of Eq (31) to vanish), the four determinants defined in Eq (30) must obey the constraint first presented in Ref. [26]:

$$\begin{array}{c} 0={\left(\Delta_{0}\right)}^{2}-\left({\left(\Delta_{x}\right)}^{2}+{\left(\Delta_{y}\right)}^{2}+{\left(\Delta_{z}\right)}^{2}\right)=\\ 1-2\left(p^{\left(1\right)}\cdot p^{\left(2\right)}\right)\left(p^{\left(1\right)}\cdot p^{\left(3\right)}\right)\left(p^{\left(2\right)}\cdot p^{\left(3\right)}\right)-{\left(p^{\left(1\right)}\cdot p^{\left(2\right)}\right)}^{2}-{\left(p^{\left(1\right)}\cdot p^{\left(3\right)}\right)}^{2}-{\left(p^{\left(2\right)}\cdot p^{\left(3\right)}\right)}^{2} \end{array}.$$ (34)

The first observation is that Eq (34) contains the (1+2)-dimensional solutions as a special case [26]. When all vectors are (1+2)-dimensional, namely, lacking *z*-components, it yields the separate vanishing of each determinant in Eq (34). In particular, Δ_*z*_ = 0. This is just the condition for the existence of (1+2)-dimensional solutions [25].

The second observation is that Eq (34) limits the choice of the three momentum vectors. Viewing it as an equation for (*p* ^(2)^ ⋅ *p* ^(3)^), the solution for the latter is:

$$\left(p^{\left(2\right)}\cdot p^{\left(3\right)}\right)=-\left(p^{\left(1\right)}\cdot p^{\left(2\right)}\right)\left(p^{\left(1\right)}\cdot p^{\left(3\right)}\right)\pm \sqrt{\left(1-{\left(p^{\left(1\right)}\cdot p^{\left(2\right)}\right)}^{2}\right)\left(1-{\left(p^{\left(1\right)}\cdot p^{\left(3\right)}\right)}^{2}\right)}\;.$$ (35)

As all the scalar products are real numbers, one can only have the following possibilities. One possibility is that the three scalar products obey *simultaneously* either Eq (19), or Eq (20). (The proof for the existence of solutions presented in [26] applies to the case of Eq (19).) Another possibility is that at least one of the three scalar products obeys

$$\left|(p^{(i)}\cdot p^{\left(j\right)})\right|=1\;.$$ (36)

Despite these limitations, Eq (34) allows for a family of three-front solutions that is much richer than the three-front solution of SG2. There are two ways to satisfy Eq (34).

1. *Δ*_0_ = 0: *Solution reduces to SG2 solution*.
  In this situation, each of the three remaining determinants on the r.h.s. of Eq (34) must vanish. As a result, the three momentum vectors must be linearly dependent. One of them, say, *p* ^(3)^, must obey Eq (32). Then, the three-front solution is a mere rotation into three space dimensions of a three-front solution of SG2. Hence again, it propagates rigidly in a plane at a velocity that is lower than, equal to, or higher than *c*, depending on whether the “basis” vectors, *p* ^(1)^ and *p* ^(2)^, obey, respectively, Eq (19) or Eq (20).
2. *Δ*_0_ ≠ 0: *Solution propagates rigidly at speed of light*.
  The geometrical interpretation provided for this case in Ref. [26] is that Eq (34) is the condition for the vanishing of the area of the hyper-surface spanned in Minkowski space by the three (1+3)-dimensional momentum vectors. This situation has, in addition, a simple physical meaning. Exploiting Eqs (29) and (30), Eq (34) can be re-written as:
  $$\beta_{x}{}^{2}+\beta_{y}{}^{2}+\beta_{z}{}^{2}=1\;.$$ (37)

Namely, the velocity of the Lorentz boost required for transforming the three-front solution to a static one (the three associated momentum vectors to be transformed to the form given by Eq (13)) is equal to speed of light. Thus, the constraint presented in Ref. [26] allows only for a non-planar three-front solution that propagates rigidly at the speed of light. This happens *despite* the fact that each individual front propagates at a velocity that is lower than *c*. The meaning of this last statement is that, if one chooses a frame of reference, in which one of the fronts is static (this is always possible), then one, or both of the other two fronts will be receding away from it at a speed that equals *c*. Such are the peculiarities of tachyonic momentum vectors…

In summary, the three-front solution of SG3 is either a space rotated three-front solution of SG2, in which case, it propagates rigidly in a plane at a velocity that is either lower than, equal to, or higher than *c*, or a genuine (1+3)-dimensional solution, which propagates rigidly at *v* = *c*.

### 4.2.2 Solutions with *N* ≥ 4 fronts

The extension to more fronts follows the steps presented in the case of SG2. Ref. [26] provides the results for *N* = 4, and the proof for any number of fronts is, again, by induction. The remaining task is the classification of the solutions. There are four subsets of solution types.

1. *Δ*_*0*_ = *0 for all momentum triplets*: *Solutions reduce to the two subsets of SG2 solutions*.
  In this case, each of the three remaining determinants on the r.h.s. of Eq (34) also vanish for all momentum triplets. As a result, only two of the momentum vectors are linearly independent, and the remaining (*N*-2) vectors obey Eq (33). The *N*-front solution is a mere rotation into three space dimensions of an *N*-front solution of SG2. Hence again, the solution propagates rigidly in a plane at a velocity that is lower than, equal to, or higher than *c*, depending on whether the “basis” vectors, *p* ^(1)^ and *p* ^(2)^, obey, respectively, Eq (19) or Eq (20).
2. *Δ*_*0*_ *≠ 0 for all triplets*.
  Solutions that belong to this subset are three-dimensional structures—branes. Each triplet of fronts propagates rigidly at *v* = *c* = 1. However, different triplets may be propagating in different directions. Hence, this is an “expanding” solution.
  The case when only three vectors are linearly independent,
  $$p^{\left(i\right)}=\mu_{i}\;p^{\left(1\right)}+\nu_{i}\;p^{\left(2\right)}+\sigma_{i}\;p^{\left(3\right)}\;\left(4\leq i\leq N\right)\;,$$ (38)
  is of particular interest. The whole set of *N* ≥ 4 fronts then propagates rigidly at the speed of light.
3. *Δ*_*0*_ *≠ 0 for some but not all triplets*: *Hybrid solutions*.
  When Eq (34) is obeyed with *Δ*_0_ ≠ 0 by some but not all momentum triplets, and with *Δ*_0_ = 0 for the remaining triplets, the solutions are also three-dimensional structures—branes. In a cluster of three fronts, for which *Δ*_0_ ≠ 0, all three momentum vectors are linearly independent. Hence, by Eqs (37) this cluster propagates rigidly at the speed of light, *c* = 1. In a cluster of three fronts, for which *Δ*_0_ = 0, only two of the three momentum vectors are linearly independent, defining a plane. Hence, this cluster may propagate at a velocity, *v* < *c*, or *v* ≥ c, depending on whether the two independent momentum vectors obey Eq (19) or (20), respectively.

Every triplet of momentum vectors has to be tested against Eqs (34) or (37). Consider one momentum vector, say, *p* ^(*i*)^. Some triplets that contain it may obey Eq (37) with *Δ*_0_ ≠ 0. Consequently, the triplets of fronts constructed from these three vectors propagates rigidly at *c* = 1. Other triplets that contain *p* ^(*i*)^ may obey Eq (37) but with *Δ*_0_ = 0. In this case, only two of the three momentum vectors in a triplet are linearly independent. Consequently, the corresponding triplet of fronts may propagate rigidly at a velocity, *v*, which obeys *v* < *c*, or *v* ≥ c, depending on whether the two independent vectors obey, respectively, Eq (19) or Eq (20).

This allows for the existence of “hybrid” solutions, in which different front clusters may have different velocities, some lower than *c*, some equal to *c* and some higher than *c*.

*Example for Hybrid solution*. To demonstrate the peculiarities of tachyonic momentum vectors, consider a four-front solution with the following momentum vectors:

$$\begin{array}{l} p^{\left(i\right)}=\left\{0,\text{cos}\;\varphi^{\left(i\right)},\text{sin}\;\varphi^{\left(i\right)},0\right\}\;,\;1\leq i\leq 3\\ p^{\left(4\right)}=\left\{p_{0}^{\left(4\right)},\text{cos}\;\varphi^{\left(4\right)},\text{sin}\;\varphi^{\left(4\right)},\pm p_{0}^{\left(4\right)}\right\}\;,\;p_{0}^{\left(4\right)}>0 \end{array}.$$ (39)

All triplets of vectors obey Eq (34). Hence, this is a valid four-front solution in (1+3) dimensions, constructed via Eqs (2)–(8). This can be also verified by direct substitution if the solution in Eq (1).

The {1,2,3} triplet obeys Eq (34) trivially, as each of the determinants in Eq (30) vanish. Consequently, the fronts constructed from *p* ^(1)^, *p* ^(2)^ and *p* ^(3)^, are static—in a rest frame in the *x*-*y* plane. However, in a triplet that contains *p* ^(4)^, Eq (34) is obeyed in a non-trivial manner; the different determinants do not vanish individually. Consequently each such triplet is seen as propagating rigidly at the speed of light.

First, consider the solution at *t* = 0. Eqs (2)–(8) generate a four-front structure, three of which, (generated from *p* ^(1)^, *p* ^(2)^ and *p* ^(3)^) lie in the *x*-*y* plane, whereas the front generated from *p* ^(4)^ protrudes outside this plane. Thus, the solution is a brane.

For *t* ≠ 0, the solution varies in time thanks to the fact that $p_{0}^{\left(4\right)}$ > 0. (The other three vectors have vanishing time components, hence, do not contribute to the time dependence of the solution.) A surprise is discovered in the limits *t* → ±∞. For *t* →-∞, the solution tends to

$$4\;{\text{tan}}^{-1}\left[\frac{e^{\xi^{\left(1\right)}}+e^{\xi^{\left(2\right)}}+e^{\xi^{\left(3\right)}}+e^{\xi^{\left(1\right)}+\xi^{\left(2\right)}+\xi^{\left(3\right)}}\;V\left(p^{\left(1\right)},p^{\left(2\right)}\right)V\left(p^{\left(1\right)},p^{\left(3\right)}\right)V\left(p^{\left(2\right)},p^{\left(3\right)}\right)}{1+e^{\xi^{\left(1\right)}+\xi^{\left(2\right)}}\;V\left(p^{\left(1\right)},p^{\left(2\right)}\right)+e^{\xi^{\left(1\right)}+\xi^{\left(3\right)}}\;V\left(p^{\left(1\right)},p^{\left(3\right)}\right)+e^{\xi^{\left(2\right)}+\xi^{\left(3\right)}}\;V\left(p^{\left(2\right)},p^{\left(3\right)}\right)}\right]\;,$$ (40)

where *ξ* ^(*i*)^ are the exponents in Eq (6):

$$\xi^{\left(i\right)}=p_{\mu }^{\left(i\right)}\;x^{\mu }\;+\delta_{i}\;,\;i=1,2,3\;.$$ (41)

As *p* ^(1)^, *p* ^(2)^ and *p* ^(3)^ have vanishing time components, the result in Eq (40) is independent of time, and represents a static three-front solution that lies in the *x*-*y* plane.

The *t* → +∞ limit of the solution is:

$$\begin{array}{c} 2\;\pi -\\ 4\;{\text{tan}}^{-1}\left[\frac{\left(\begin{array}{l} e^{\xi^{\left(1\right)}}\;V\left(p^{\left(1\right)},p^{\left(4\right)}\right)+e^{\xi^{\left(2\right)}}\;V\left(p^{\left(2\right)},p^{\left(4\right)}\right)+e^{\xi^{\left(3\right)}}\;V\left(p^{\left(3\right)},p^{\left(4\right)}\right)+\\ e^{\xi^{\left(1\right)}+\xi^{\left(2\right)}+\xi^{\left(3\right)}}\;V\left(p^{\left(1\right)},p^{\left(2\right)}\right)V\left(p^{\left(1\right)},p^{\left(3\right)}\right)V\left(p^{\left(1\right)},p^{\left(4\right)}\right)V\left(p^{\left(2\right)},p^{\left(3\right)}\right)V\left(p^{\left(2\right)},p^{\left(4\right)}\right)V\left(p^{\left(3\right)},p^{\left(4\right)}\right) \end{array}\right)}{\left(\begin{array}{l} 1+e^{\xi^{\left(1\right)}+\xi^{\left(2\right)}}\;V\left(p^{\left(1\right)},p^{\left(2\right)}\right)V\left(p^{\left(1\right)},p^{\left(4\right)}\right)V\left(p^{\left(2\right)},p^{\left(4\right)}\right)\\ +e^{\xi^{\left(1\right)}+\xi^{\left(3\right)}}\;V\left(p^{\left(1\right)},p^{\left(3\right)}\right)V\left(p^{\left(1\right)},p^{\left(4\right)}\right)V\left(p^{\left(3\right)},p^{\left(4\right)}\right)\\ +e^{\xi^{\left(2\right)}+\xi^{\left(3\right)}}\;V\left(p^{\left(2\right)},p^{\left(3\right)}\right)V\left(p^{\left(2\right)},p^{\left(4\right)}\right)V\left(p^{\left(3\right)},p^{\left(4\right)}\right) \end{array}\right)}\right] \end{array}.$$ (42)

This is also a static solution, with the same three fronts. Relative to the *t* →-∞ limit, they are shifted by finite shifts in the *x*-*y* plane. The shifts are consequences of the numerical coefficients that multiply each of the exponential functions. In addition, the sign of each front is flipped.

Now consider a Lorentz transformation along the *z*-axis. In the limit of the boost velocity approaching *c* (corresponding to a reference frame that moves along the *z*-axis at a velocity equal to ±*c* (*z* = ±*t*, the sign corresponding to $\pm p_{0}^{\left(4\right)}$ in Eq (39)), the solution tends to a static four-front structure; the four fronts are seen as propagating rigidly at the speed of light.

## Invariance Property of Slower-Than-Light Solutions in (1+3) Dimensions

The slower-than-light (1+3)-dimensional *N*-front solutions of SG3 deserve additional attention. Only two of the *N* momentum vectors are linearly independent. Consider, first, a static solution. The space components of all the momentum vectors (${\vec{q}}^{\left(i\right)}$ of Eq (13)) lie in a plane in the three-dimensional space. Denote the unit vector normal to this plane by $\vec{n}$. In a moving frame, ${\vec{q}}^{\left(i\right)}$ are transformed to the (1+3) dimensional space-like momentum vectors *p* ^(*i*)^, and $\vec{n}$ is transformed into a space-like vector, *n*, obeying

$$n_{\mu }\;p^{\left(i\right)}{}^{\mu }=0\;,\;p^{\left(i\right)}{}_{\mu }p^{\left(i\right)}{}^{\;\mu }=n_{\mu }\;n^{\mu }=-1\;.$$ (43)

As the *x*-dependence of the solution appears only through the Lorentz invariant scalar product in Eq (6), the front solutions in (1+3) dimensions are invariant under the transformation:

u(x;Q)=u(x+α(x)n;Q), (44)

for any scalar function *α*(*x*). In particular, Eq (44) implies that the current density obeys:

$$n^{\mu }J_{\mu }=n^{\mu }\partial_{\mu }u=0\;.$$ (45)

## Numerical examples

The solutions of physical interest are the slower-than-light ones. These are two-dimensional structures, and can be always Lorentz-transformed to a rest frame, where they are static. Therefore, the examples shown here are of static solutions over two space coordinates. The space part of each (1+2)-dimensional momentum vector used in the construction of the solution through Eqs (2)–(8) is given by:

$${\vec{q}}^{\left(i\right)}=\{\text{cos}\;\varphi_{i},\text{sin}\;\varphi_{i}\}\;.$$ (46)

**Fig 1:**
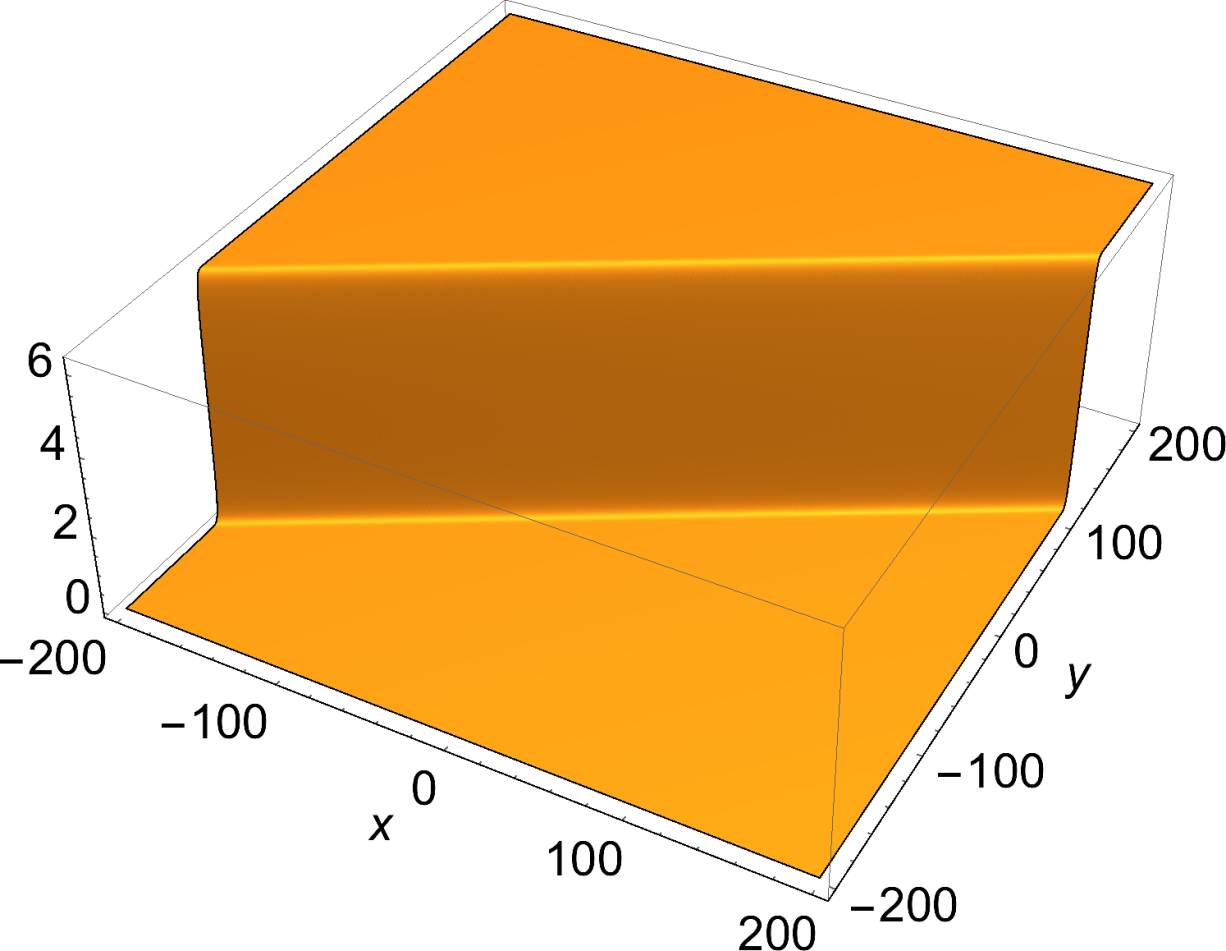
Static single-front solution. Momentum vector given by Eqs (13) and (46). *φ*_1_ = -*π*/3, *δ*_1_ = 0.

**Fig 2:**
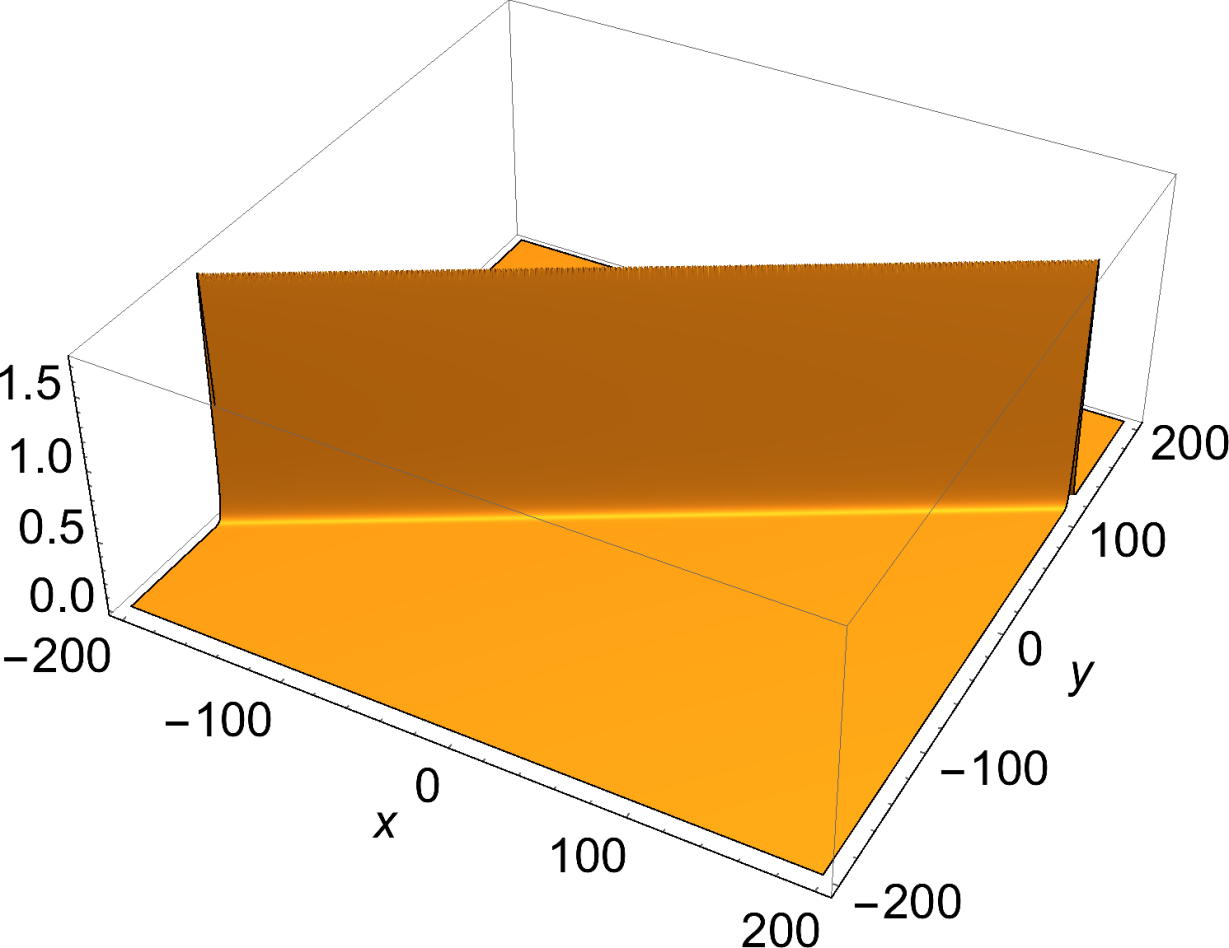
*y*-component of current density (Eq (9)) of single-front solution of Fig 1.

**Fig 3:**
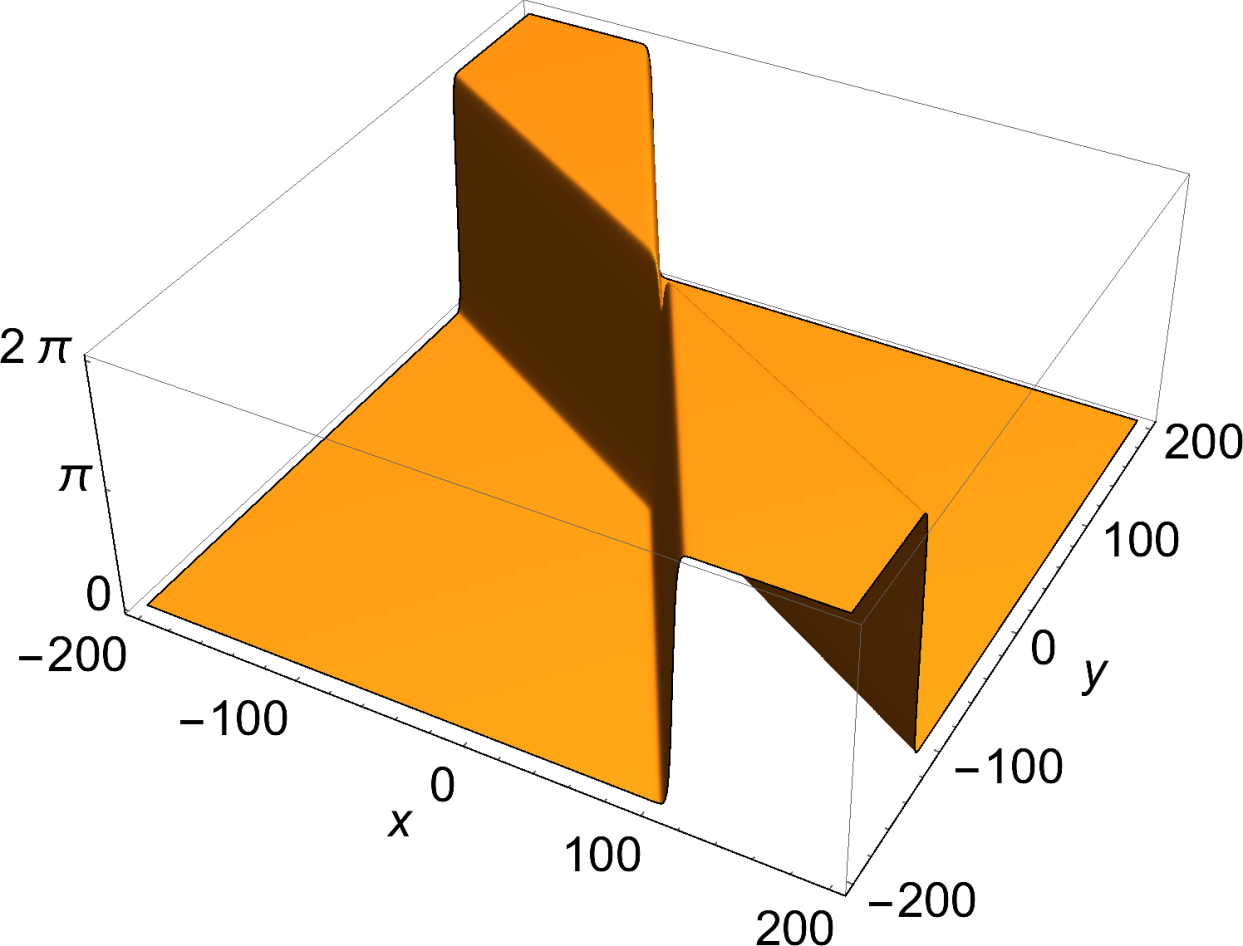
Static two-front solution. *φ*_1_ = *π*/6, *φ*_2_ = *π*/3, *δ*_1_ = *δ*_2_ = 0.

**Fig 4:**
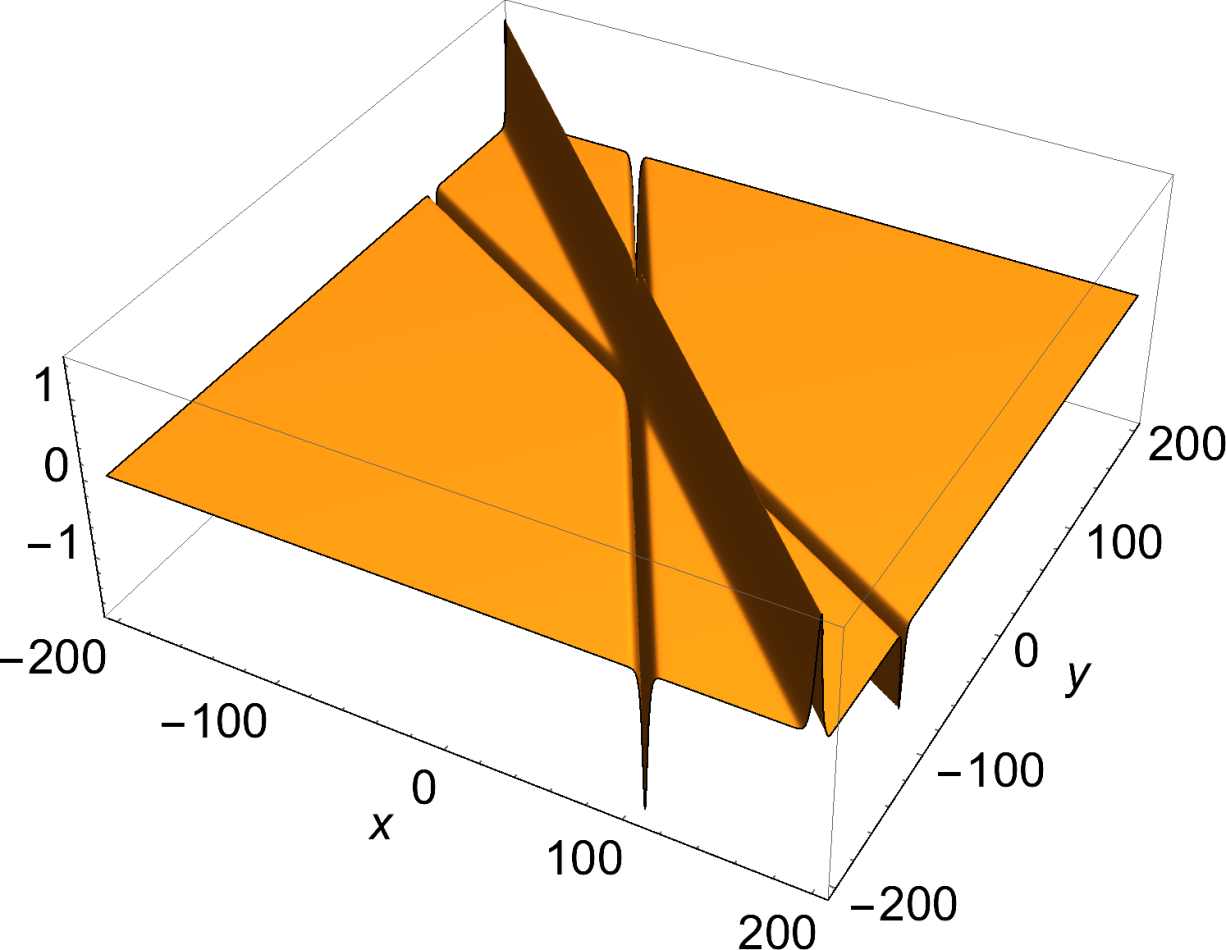
*x*-component of current density (Eq (9)) of three-front solution. *φ*_1_ = *π*/4, *φ*_2_ = *π*/3, *φ*_3_ = -*π*/4, *φ*_4_ = -π/3, *δ*_1_ = *δ*_2_ = *δ*_3_ = *δ*_4_ = 0.

**Fig 5:**
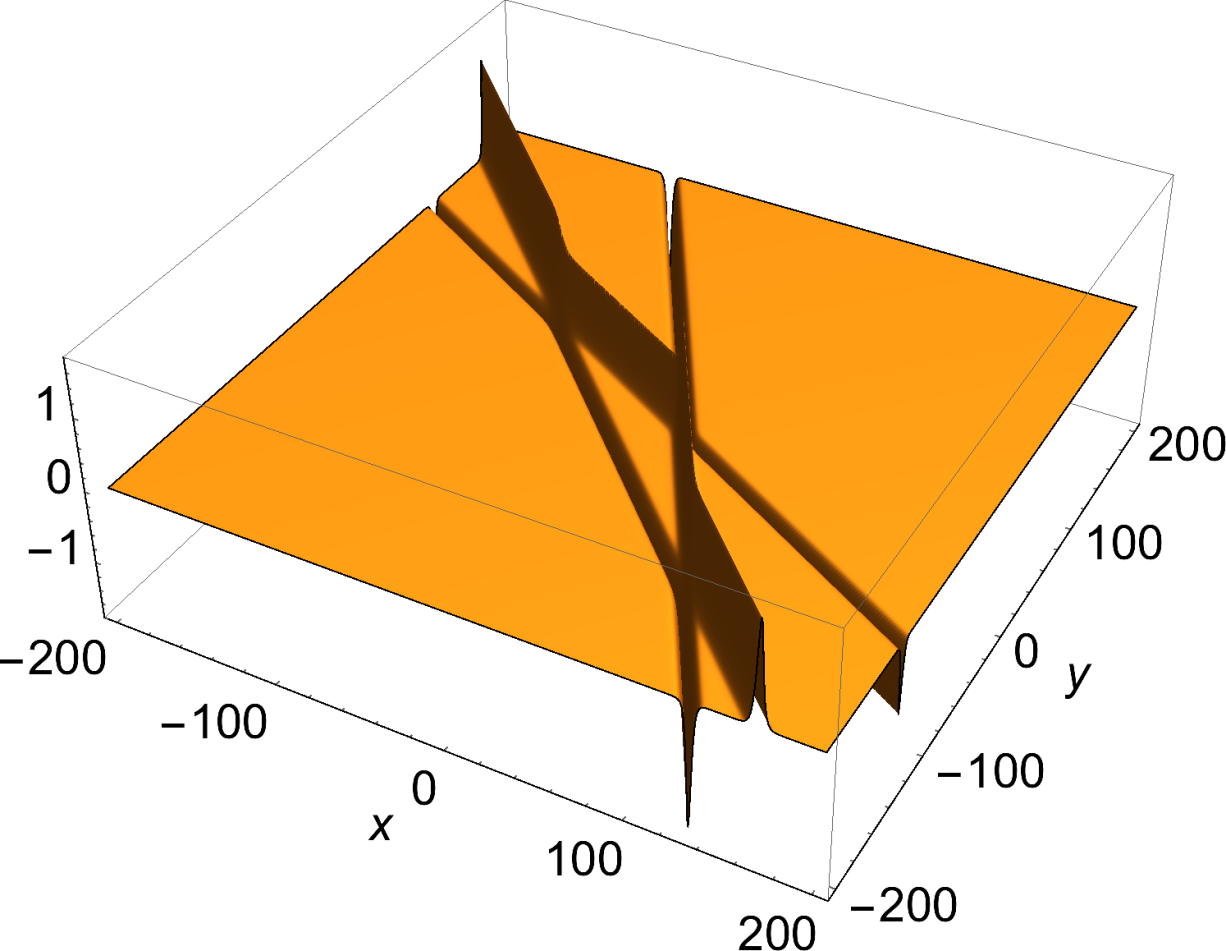
*x*-component of current density (Eq (9)) of three-front solution. *φ*_1_ = *π*/6, *φ*_2_ = *π*/3, *φ*_3_ = *π*/4, *δ*_1_ = 20, *δ*_2_ = 0, *δ*_3_ = -20.

**Fig 6:**
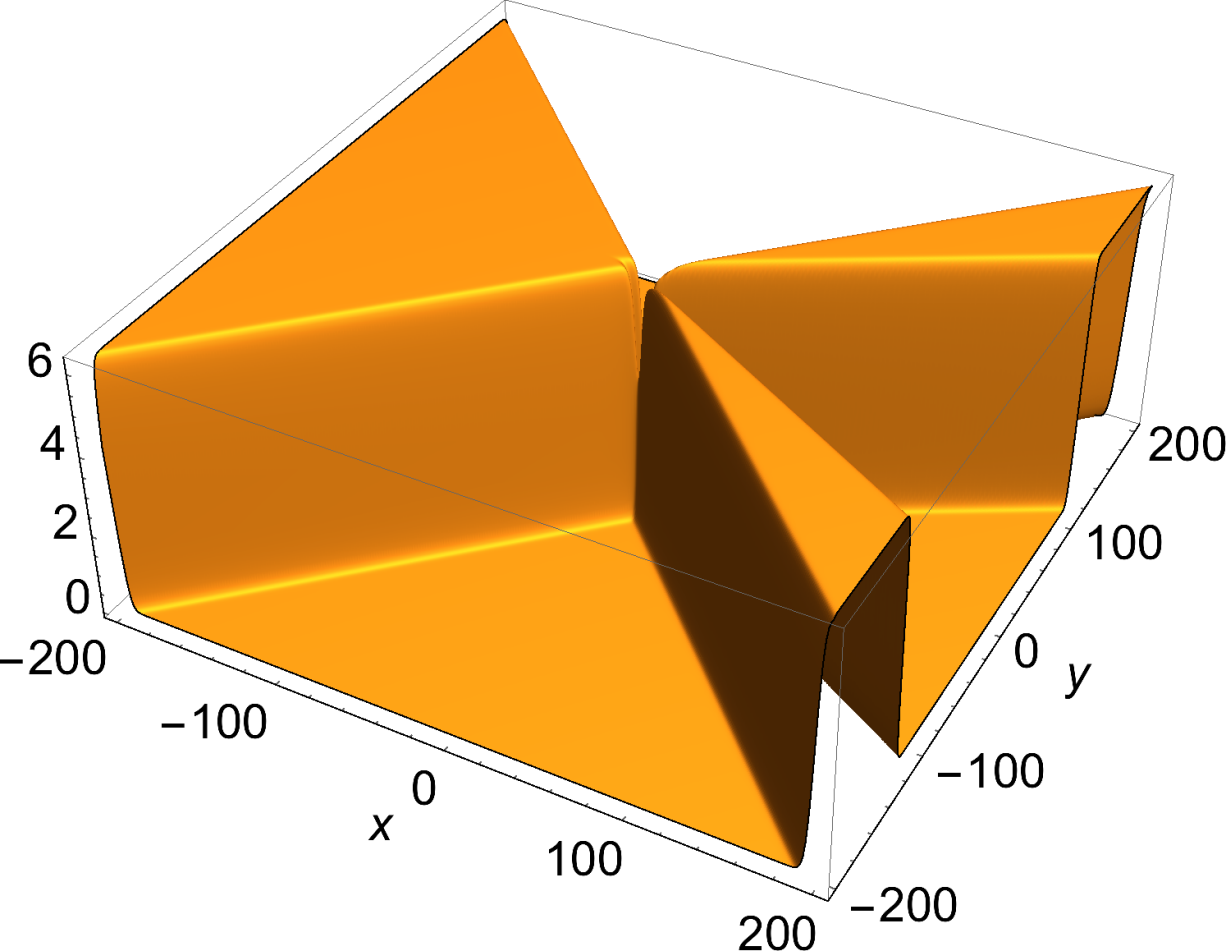
Static four-front solution. *φ*_1_ = *π*/6, *φ*_2_ = *π*/3, *δ*_1_ = *δ*_2_ = 0.

Fig 1 shows the single-front solution. Fig 2 shows *J*_*y*_ = *∂*_*y*_ *u*, the *y*-component of the current density, in which a single soliton is visible. Fig 3 shows a two-front solution. To show the effect of the choice of the free phases in Eq (6) on the number of junctions in a solution, Figs 4 and 5 show *J*_*x*_ = *∂*_*x*_ *u*, the *x*-component of the current density of a three-front solution for, respectively, a case of a single junction and a case of three junctions. The current density has been chosen rather than the solution, because the junctions are easier to discern in that plot. Fig 6 presents a four-front solution.

## The Case of Time-Like Momentum Vectors

Consider the following modification of Eq (1):

$$\partial_{\mu }\partial^{\mu }u-\text{sin}\;u=0,\;\mu =0,1,..,n,\;n=1,2,3\;.$$ (47)

A trivial way to obtain Eq (47) is to replace *u* by (*u* ± *π*) in Eq (1). One constructs the solutions in the manner described in Section 1.2.1, and then adds ± *π* to the result. However, unlike the solutions of Eq (1), the resulting solutions of Eq (47) then do not vanish at infinity in some direction in the (1+*n*)-dimensional space. The following discussion addresses the non-trivial case, in which front solutions of Eq (47) obey vanishing boundary conditions in some direction at infinity. The application of the Hirota algorithm generates *N*-front solutions for all *N* ≥ 1, for *n* = 1,2,3. The only changes in the algorithm are that Eqs (7) and (8) are replaced by:

$$p^{\left(i\right)}{}_{\mu }\;p^{\left(i\right)\;\mu }=+1\;,$$ (48)

and

$$V\left(p,p'\right)=\frac{1-p_{\mu }\;p{'}^{\mu }}{1+p_{\mu }\;p{'}^{\mu }}\;.$$ (49)

The constraints for the existence of *N* ≥ 3 front solutions in (1+2) dimensions, (Eq (33)), and in (1+3) dimensions (Eq (34)), are arrived at also in the present case.

Owing to Eq (48), in all space dimensions, an individual front (be it the single-front solution, or one front in a multi-front solution) propagates at a velocity, *v* ≥ *c*. The (1+1)-dimensional solutions are readily constructed. Interesting effects of the time-like nature of the momentum vectors on multi-front solutions show up in higher space dimensions. The effects are consequences of the fact that there is no Lorentz transformation that can simultaneously transform two, or more, *different* time-like vectors to a rest frame (certainly, no transformation to Eq (13) exists!).

*(1+2) dimensions*: The fronts in a two-front solution propagate each at a different velocity, *v* ≥ *c*. In solutions with *N* ≥ 3 fronts, Δ_*z*_, defined in Eq (30), must vanish for all triplets of momentum vectors. Hence, only two vectors are linearly independent, and all remaining vectors must obey Eq (33). Again, each front propagates at a different velocity, *v* ≥ *c*.

*(1+3) dimensions*: *Two-front solution*: The fronts in a two-front solution propagate each at a different velocity, *v* ≥ *c*. Hence, this solution is a rotated (1+2)-dimensional solution.

*N-front solutions*, *N ≥ 3* : Unlike the case of space-like momentum vectors, analyzed in the preceding sections, all (1+3)-dimensional solutions with *N* ≥ 3 fronts are also mere space rotations of (1+2)-dimensional solutions; all momentum-vector triplets must obey Eq (33). Namely, there are only two linearly independent vectors. This characteristic is a direct consequence of two properties of the solutions. The first property is the time-like nature of the momentum vectors (Eq (48)). The second property is that all momentum triplets must obey Eq (34), with Δ_0_, Δ_*x*_, Δ_*y*_ and Δ_*z*_ defined in Eq (31). (This last statement is reached by a repetition of the analysis of Ref. [26].)

To see that all momentum-vector triplets must obey Eq (33), consider a triplet. One can always Lorentz transform one of the momenta, say *p* ^(3)^, to its rest frame:

$$p^{\left(3\right)}\to \left\{1,0,0,0\right\}\;.$$ (50)

As a result, Δ_0_ of Eq (30) vanishes in the rest frame. Obeying Eq (34) then requires that each of the three determinants, Δ_*x*_, Δ_*y*_ and Δ_*z*_ must vanish. This, in turn, requires that the space parts of *p* ^(1)^ and *p* ^(2)^ must be proportional to one another:

$$p^{\left(1\right)}=\left\{p_{0}^{\left(1\right)},{\vec{p}}^{\left(1\right)}\right\}\;,\;p^{\left(2\right)}=\left\{p_{0}^{\left(2\right)},\alpha {\vec{p}}^{\left(1\right)}\right\}\;.$$ (51)

Eqs (48), (50) and (51) yield a linear relation amongst the (1+3)-dimensional vectors:

$$p^{\left(3\right)}=\frac{\left(\alpha \;p^{\left(1\right)}-p^{\left(2\right)}\right)}{\alpha \;p_{0}^{\left(1\right)}-p_{0}^{\left(2\right)}}\;.$$ (52)

This linear relation is preserved when the three vectors are transformed back to their forms prior to the transformation that yields Eq (50). As this conclusion applies to all momentum triplets, only two of the momenta are linearly independent. Hence, all *N*-front solutions in (1+3) dimensions are merely space-rotated (1+2)-dimensional solutions.

## Concluding Comments

Despite the fact that the Sine-Gordon equation in (1+2) and (1+3) dimensions does not pass traditional integrability tests, it does have a wealth of single- and multi-front solutions with interesting physical characteristics. These characteristics are, to a great extent, consequences of properties of the tachyonic momentum vectors, from which the solutions are constructed, under Lorentz transformations in Minkowski space. Some of these characteristics defy our intuition, which is based on our experience with time-like momentum vectors, vectors that represent physical particles.

Of particular significance are the characteristics of solutions that propagate rigidly at velocities that are lower than the speed of light, *c* = 1. They exist in (1+2) dimensions, as well in (1+3) dimensions, where they are merely space-rotated (1+2) dimensional solutions, so that they have a planar configuration. The subspace of slower-than-light solutions cannot be connected by a continuous change of parameters into the subspace of solutions that have faster-than-light front clusters. This is, clearly a consequence of the fact that the solutions of Eq (1), constructed through Eqs (2)–(8), are scalars under Lorentz transformations, and that a relativistically invariant system does not allow the crossing of the speed of light from physical systems that are slower than light.
